# Formulation Considerations for the Management of Dry Eye Disease

**DOI:** 10.3390/pharmaceutics13020207

**Published:** 2021-02-03

**Authors:** Priyanka Agarwal, Jennifer P. Craig, Ilva D. Rupenthal

**Affiliations:** 1 Buchanan Ocular Therapeutics Unit, Department of Ophthalmology, New Zealand National Eye Centre, The University of Auckland, Auckland 1142, New Zealand; p.agarwal@auckland.ac.nz; 2 Department of Ophthalmology, New Zealand National Eye Centre, Faculty of Medical and Health Sciences, The University of Auckland, Auckland 1142, New Zealand; jp.craig@auckland.ac.nz

**Keywords:** dry eye disease, ocular drug delivery, artificial tears, cyclosporine A

## Abstract

Dry eye disease (DED) is one of the most common ocular surface disorders characterised by a deficiency in quality and/or quantity of the tear fluid. Due to its multifactorial nature involving several inter-related underlying pathologies, it can rapidly accelerate to become a chronic refractory condition. Therefore, several therapeutic interventions are often simultaneously recommended to manage DED efficiently. Typically, artificial tear supplements are the first line of treatment, followed by topical application of medicated eyedrops. However, the bioavailability of topical eyedrops is generally low as the well-developed protective mechanisms of the eye ensure their rapid clearance from the precorneal space, thus limiting ocular penetration of the incorporated drug. Moreover, excipients commonly used in eyedrops can potentially exhibit ocular toxicity and further exacerbate the signs and symptoms of DED. Therefore, formulation development of topical eyedrops is rather challenging. This review highlights the challenges typically faced in eyedrop development, in particular, those intended for the management of DED. Firstly, various artificial tear supplements currently on the market, their mechanisms of action, as well as their application, are discussed. Furthermore, formulation strategies generally used to enhance ocular drug delivery, their advantages and limitations, as well as their application in commercially available DED eyedrops are described.

## 1. Introduction

Dry eye disease (DED) is one of the most prevalent ocular surface disorders affecting tens of millions of individuals globally [1]. Recently, the Definition and Classification Subcommittee of the second Tear Film and Ocular Surface Society Dry Eye Workshop (TFOS DEWS II) reviewed the existing literature to achieve an international consensus on the current working knowledge of DED and defined it as “a multifactorial disease of the ocular surface characterised by a loss of homeostasis of the tear film, and accompanied by ocular symptoms, in which tear film instability and hyperosmolarity, ocular surface inflammation and damage, and neurosensory abnormalities play aetiological roles” [2]. Although DED is not sight-threatening in most patients, visual impairment associated with low spatial frequency and contrast sensitivity, increased glare, blurred vision and eye fatigue are frequently reported [3,4,5]. In addition to these direct pathological manifestations, DED also tends to compromise physical functioning, social interaction and general health and well-being of patients, resulting in a significant deterioration in their quality of life [6,7,8].

DED is often classified into two primary subtypes: aqueous tear-deficient dry eye (ADDE), characterised by inefficiency or inability of the lacrimal glands to produce tears, and evaporative dry eye (EDE), typically attributed to excessive evaporation of the tear fluid. ADDE may or may not have an autoimmune origin and is generally attributed to a compromise in the integrity of the lacrimal functional unit. EDE is the more common form of DED and is frequently associated with meibomian gland dysfunction (MGD) characterised by modification or reduction of tear fluid lipids, due to which, integrity and quality of the tear fluid may be compromised [9,10]. A number of pathological mechanisms, including inflammation, microbial contamination and lipid deficiencies can trigger MGD [11]. Although traditionally, DED has been classified into these two subtypes, it is acknowledged that there is considerable overlap between them [2]. As such, chronic conditions are most often characterised by a “hybrid” or “mixed” form of the disease, wherein each DED subtype eventually adopts some clinical features of the other [12,13]. It is now understood that the various DED pathologies are not exclusive of each other, but rather, they tend to initiate and exacerbate each other forming a “vicious circle” of DED and MGD [2,14]. Consequently, multiple therapeutic strategies are often employed simultaneously to target DED pathologies with topically applied tear supplements typically being the first line of intervention.

Topical application, being simple, convenient, and painless, is the preferred route for administration of prescription drugs to treat ocular surface conditions as it reduces systemic side effects by localising the drug close to the target site. Moreover, for a number of drugs, it is the only means of achieving therapeutic concentrations in the eye, since the blood-aqueous barrier otherwise prevents systemically administered drugs from reaching anterior segment tissues [15]. Not surprisingly, over 90% of ophthalmic formulations currently on the market are topical eyedrops [16]. However, the efficacy of topically applied formulations is limited by the various protective mechanisms of the eye which reduce drug bioavailability, thus necessitating frequent eyedrop administration over prolonged periods. This in turn is often associated with reduced patient compliance further limiting treatment efficacy. Concomitant administration of multiple eyedrops, as is often necessary to manage DED, may further complicate the treatment regimen and reduce compliance. This review discusses the challenges encountered in developing topical formulations, specifically highlighting those attenuated by the ocular surface compromise typically observed in DED. Additionally, formulations generally used in the management of DED to target the different underlying pathologies, their postulated benefits and their formulation characteristics are also discussed.

## 2. Formulation Challenges

### 2.1. Rapid Precorneal Clearance

The dynamic nature of the ocular surface results in rapid clearance of foreign substances from the eye due to blinking, nasolacrimal drainage and reflex and basal tearing. The conjunctival sac, which serves as a reservoir for topically applied formulations, has a volume of approximately 7–8 µL and can distend to a maximum capacity of 30 µL without blinking [17]. Meanwhile, eyedrops instilled with commercial droppers typically have a volume of 40 µL or more [18]. The eye attempts to achieve homeostasis immediately after eyedrop instillation by reflex blinking and tearing to expel foreign substances and restore the normal tear volume, which results in immediate overflow and expulsion of excess fluid [17,19]. It has been estimated that less than 10 µL of the applied dose remains on the ocular surface following a single blink, leaving a short window of approximately 5–7 min for drug absorption, especially when the rapid tear fluid turnover (19.7 ± 6.5%/min) is taken into account [20].

Concomitant administration of two or more eyedrops, as is often necessary for DED, can further reduce precorneal residence time and ocular bioavailability by increasing competition for volume in the precorneal space [21], with the time interval between eyedrop administration negatively correlating with corneal bioavailability [22,23]. On the other hand, corneal drug concentration post-administration of a single eyedrop formulation containing two drugs is reportedly similar to that observed after administration of eyedrops containing equivalent amounts of each drug, individually [22]. Thus, combination eyedrop formulations capable of simultaneously treating more than one of the underlying DED pathologies could potentially improve the ocular bioavailability and treatment efficacy.

### 2.2. Poor Drug Penetration

**Figure 1:**
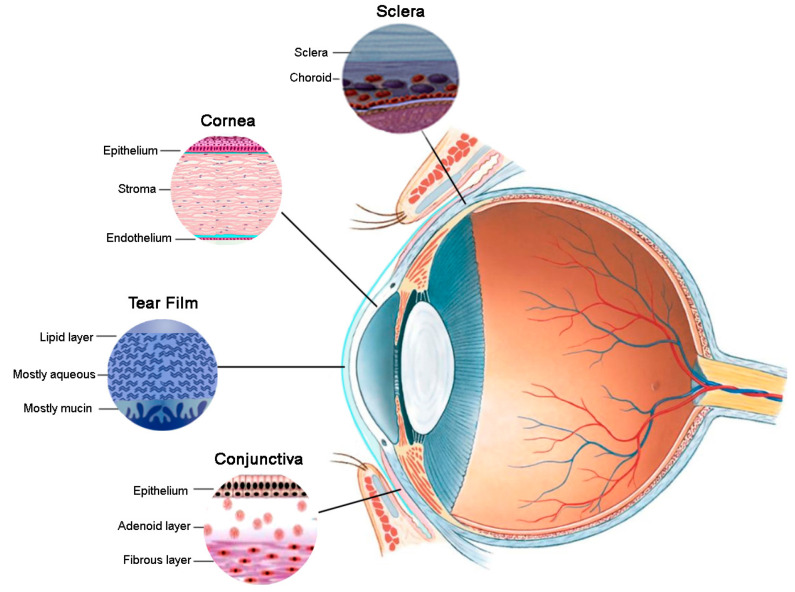
Penetration barriers to topical drug delivery.

In addition to the rapid clearance of topically applied medications from the ocular surface, the ocular bioavailability of drugs from medicated eyedrops is further limited by the nature of the tear fluid and ocular tissues, which together pose a formidable barrier to intraocular transport of drugs (Figure 1).

The tear film is the eye’s first line of defence with its various components working synergistically to minimise exposure to foreign substances. Superficially, it comprises a thin lipid layer which limits access of aqueous formulations to the corneal interface while also minimising excessive tear evaporation. Underlying the lipid layer is the aqueous phase of the tear film, rich in enzymes, proteins, and mucins that can inactivate drugs by protein binding or enzymatic degradation, and thus reduce their bioavailability [24]. The region of the aqueous layer closest to the goblet cells is the most concentrated in mucins which can entrap drug particles by the formation of low affinity polyvalent adhesive interactions, rapidly eliminating them from the ocular surface [25,26].

The cornea is the most anterior ocular tissue and consists of alternating hydrophobic and hydrophilic layers. The hydrophobic corneal epithelium is the major barrier to drug transport. It is composed of 5–7 layers of epithelial cells with tight intercellular junctions, therefore, only very small molecules can traverse paracellularly through the cornea. Transcellular transport, on the other hand, is generally only possible for smaller molecular weight lipophilic drugs [27]. Underlying the hydrophobic epithelium is the hydrophilic stroma which favours the penetration of low molecular weight hydrophilic drugs while hindering the passage of lipophilic drugs. Therefore, hydrophobic drugs tend to be retained in the corneal epithelium from where they are released very slowly into the posterior tissues [28]. Overall, it has been estimated that less than 10% of a topically applied dose reaches the intraocular environment through the cornea [29].

While traditionally not considered a major drug delivery route, ocular drug penetration may also occur via the conjunctival-scleral pathway which provides a much larger surface area for drug absorption than the cornea [28]. The conjunctival epithelium is relatively leaky and hydrophilic, with intercellular spaces being approximately 230-fold larger than those in the cornea, rendering it permeable even to large biomolecules, such as proteins and peptides [30,31]. The conjunctival epithelium is more permeable to hydrophilic drugs with the permeability of hydrophilic polyethylene glycol mixtures reportedly being the highest in the conjunctiva, followed by the sclera and cornea, respectively [31]. However, since the sclera and conjunctiva are richly perfused by blood vessels, a large fraction of drug absorbed via this route may be lost to the systemic circulation [32].

### 2.3. Dose Volume

Due to limited precorneal space, a smaller eyedrop volume (5–15 µL) is preferable to minimise drug wastage and reduce the risk of systemic toxicity. The dose-volume can be controlled to some extent by training the patient in eyedrop administration and by modifying the dropper tip and angle [33,34]. Piezoelectric micro-dosing systems have also been developed to consistently deliver a very small eyedrop volume [35]; however, these devices are rather expensive. Physical characteristics of the formulation, such as surface tension, cohesive forces, viscosity and density can also influence the drop size [36]. For instance, in situ gelling systems, such as hydroxypropyl-guar Systane^®^, by virtue of their lower viscosity, reduce dosing errors in comparison to viscous gels [37]. Surfactants and penetration enhancers, such as tetracaine, polysorbate 80 and benzalkonium chloride (BAK), can also reduce the drop size to some extent by reducing the surface tension of the formulation [33]; however, due to the toxicity typically associated with these excipients, their inclusion is rarely justified for the purpose of reducing drop size alone. Certain non-aqueous liquids, such as semifluorinated alkanes (SFAs), which inherently have lower surface tension and viscosity than aqueous eyedrops, may help in achieving a smaller drop size and minimise overflow [38].

### 2.4. Visual Disturbance

Transiently reduced visual acuity post-instillation is another limitation of eyedrops that correlates with their viscosity and refractive index. For example, mid-viscosity Refresh Liquigel^®^ can cause more blurring than low viscosity Refresh Tears^®^ [39]. Blurring of vision is also commonly reported with in situ gelling systems, likely due to a sudden change in viscosity post-instillation [40]. To minimise visual disturbance, topically applied eyedrops should be optically transparent and ideally have a refractive index identical to that of the tear fluid (1.336–1.338) [41]. Nevertheless, the refractive index of most formulations currently on the market is relatively high (oily eyedrops typically have a refractive index of 1.44–1.50), resulting in frequent complaints of blurred vision and foreign body sensation [42].

### 2.5. Preservative Toxicity

Several experimental and clinical studies have demonstrated that most preservatives used in ophthalmic formulations have pronounced ocular toxicity. BAK, the most commonly used preservative in topical eyedrops, has repeatedly been shown to be toxic to the ocular surface, leading to exacerbated DED symptoms [43,44]. BAK disrupts the integrity of corneal tight junctions which may compromise its barrier properties and elevate the toxicity potential of other drugs and excipients. Therefore, eyedrops preserved with BAK not only have a direct detrimental effect on the ocular surface but may also potentiate the toxicity of other excipients in the same formulation or those applied concomitantly. In fact, one study has suggested that with each additional dose, eyedrops containing BAK increase the risk of ocular surface disease two-fold [45]. However, despite the overwhelming evidence of its toxicity, a number of products containing BAK remain commercially available and are not infrequently used in the treatment of DED [46].

Significant ocular toxicity has also been associated with other antimicrobial agents used in eyedrops, including parabens, sodium perborate, chlorobutanol, stabilised thiomersal, and ethylenediamine tetraacetic acid (EDTA) [47]. Meanwhile, the cytotoxic effect of newer generation preservatives, such as Polyquad^®^, Purite^®^_,_ and SofZia^®^, is comparatively low [48], although their long-term effect on tear film stability is currently unknown. It should be noted that the toxicity of preservatives may incidentally be enhanced by viscosity-building agents present in eyedrops. For instance, corneal epithelial damage has been observed when the thickening agent hydroxyethylcellulose is used with BAK, although no such effect was observed when either excipient was used alone [49]. Similarly, punctal plugs, commonly used in DED therapy to reduce tear drainage, can increase the exposure to toxic preservatives enhancing their detrimental effects.

To enable the delivery of preservative-free eyedrops to the ocular surface, preparations may be supplied in single-dose units; however, such eyedrops can cost 5–10 times more than multidose formulations and are often difficult to handle [50]. Multidose preservative-free dosing systems have thus been developed to overcome these limitations. One such dosing system is the third generation ABAK^®^ bottle (Théa Laboratories, Clermont-Ferrand, France) which uses a bi-functional membrane with antimicrobial properties to maintain sterility for up to three months after opening. Sterile filters are also used in the Clear Eyes^®^ bottle (Prestige Consumer Healthcare, Greenburgh, NY, USA) and the hydraSENSE^®^ delivery system (Bayer, Leverkusen, Germany), while the COMOD^®^ dosage system (Ursapharm, Saarbrücken, Germany) uses a one-way valve to maintain sterility for up to six months after opening [46]. Although these systems reduce the difficulties associated with handling single-dose products, their cost remains significantly higher than that of conventionally preserved eyedrops.

### 2.6. Poor Tolerability of Formulation Excipients

In view of recent clinical experience and literature evidence, the TFOS DEWS II Iatrogenic Subcommittee listed several formulation excipients, including surfactants, pH modifiers and antioxidants, in addition to preservatives, as agents with the potential to cause DED [51]. However, almost all of these compounds are commonly found in over-the-counter artificial tear supplements and DED medications currently on the market. An increased incidence of local adverse effects, such as stinging, burning and excessive tearing, has been reported due to high surfactant concentrations in topical formulations. The risk of toxicity is particularly high in novel colloidal drug delivery systems, such as micelles, micro- or nanoemulsions, liposomes and nanoparticles, due to the higher proportion of surfactants and co-surfactants used compared to conventional formulations [52,53]. Surfactants and co-surfactants can further destabilise the tear film exacerbating DED symptoms [51,54]. Consequently, iatrogenic ocular surface disease, caused by “commission” rather than the “omission” of treatment, is a significant concern with eyedrops.

As discussed earlier, excipient toxicity too may be exacerbated by concomitant administration of multiple eyedrops. For example, Restasis^®^ and Refresh^®^ Endura artificial tear supplements both contain polysorbate 80, which can reportedly trigger DED [51]. However, these eyedrops are frequently recommended in combination for DED therapy [55] and this practice may significantly increase the toxicity potential by increasing the overall exposure. Finally, adverse effects may also become more pronounced on exposure to multiple iatrogenic excipients (in addition to preservatives) simultaneously.

### 2.7. Poor Patient Compliance

Non-compliance with treatment regimens is one of the biggest challenges in treating ocular surface disorders. In a phone survey performed in 239 patients [56], 37–53% of patients with prescribed topical eyedrops had discontinued use. Inter-day and inter-individual variability appeared to be high with most patients arbitrarily titrating the dose to the severity of symptoms on any given day. The chief reasons for discontinuing treatment were reportedly failure to purchase a new dosage unit followed by failure to perceive any improvement [56]. In DED, therapeutic outcomes generally become evident only after long-term treatment. Reduced corneal sensitivity in late stages of the disease may also result in failure to notice any change in symptoms, which ironically results in worsening of patient compliance as the severity of the disease progresses [57]. Adverse effects associated with eyedrops can also reduce the patient’s willingness to continue the treatment. The absence of adverse effects is reportedly one of the most desirable product characteristics from the patients’ perspective with almost 85% of patients willing to pay more for eyedrops that have fewer adverse effects [58,59].

DED therapy usually involves frequent administration of eyedrops over a prolonged period of time, which further reduces patient compliance. Socioeconomic studies analysing patients’ responses to eyedrops have shown that patient preference increases as the dosing frequency reduces [58]. For many patients, adding a second eyedrop adversely affects compliance by increasing the complexity of the dosing regimen, potentially also increasing dosing errors [60,61]. Meanwhile, patient preference analysis using a “willing-to-pay” questionnaire has shown that patients strongly prefer combination eyedrops which can be administered from a single bottle over having to use multiple eyedrops [58].

The long-term financial burden of DED treatment is another major reason for poor patient compliance. The annual cost of DED treatment in the US in 2008 was estimated to range between USD 438–2964 per patient, depending upon the severity of the disease, the recommended treatment plan and the number of specialist visits, with the acquisition of Restasis eyedrops being the single most significant contributor to the treatment cost [57].

## 3. Management of Dry Eye Disease

**Figure 2:**
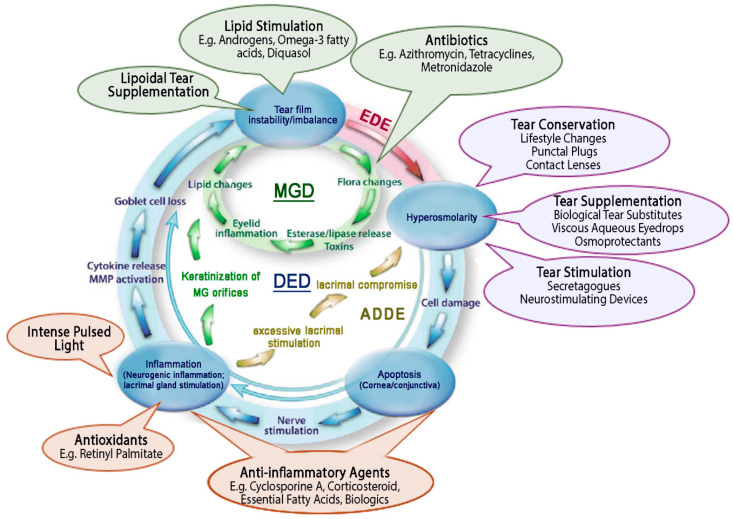
Therapeutic management strategies for dry eye disease (DED). Adapted with permission from [14]; Published by Elsevier, 2013.

The eye is a complex and dynamic organ with robust homeostatic mechanisms designed to optimise its function. Disruption to the homeostasis of the ocular surface leads to multiple downstream effects resulting in the development of a self-sustained vicious circle of DED pathologies. Therefore, concurrent management of the different underlying pathologies, such as hyperosmolarity, apoptosis and inflammation, as well as tear film instability (Figure 2), is desirable [10,62].

Selecting therapeutic interventions based on disease severity is generally recommended to ensure that desirable effects of the intervention outweigh undesirable adverse events (Table 1). Moderate lifestyle changes along with artificial tear supplementation are generally adequate in mild or preclinical DED (Grade I). Prescription drugs for pathogenic treatment may additionally be needed for management of moderate to severe conditions (Grades 2 and 3). Surgical intervention is considered only if these treatment recommendations fail to yield any positive results (Grade 4).

Topically applied aqueous and/or lipid-based tear supplements, along with lid hygiene, are the mainstay of DED therapy [64]. The next line of treatment generally involves medicated topical eyedrops containing antibiotics and/or anti-inflammatory agents, corticosteroids, omega-3 fatty acids or tear secretagogues, as further discussed below.

### 3.1. Tear Supplementation

#### 3.1.1. Aqueous Tear Supplementation

Aqueous tear supplementation is often the first line of treatment comprising either isotonic or hypotonic aqueous eyedrops which typically function by augmenting the aqueous layer and transiently reducing tear fluid osmolarity. Thus, osmolarity balanced artificial tear supplements are a preferred treatment in individuals with low baseline tear volume [65]. Artificial tear supplements with an electrolyte balance similar to human tears, such as Conheal™ eyedrops (Pannon Pharma Ltd., Pécsvárad, Hungary) and BION Tears^®^ (Alcon, Fort Worth, TX, USA), can improve corneal integrity [66,67].

Hypotonic tear substitutes are believed to be more efficient in ameliorating dry eye symptoms [68]; however, their effect is generally short-lived due to rapid clearance from the eye. Consequently, baseline tear osmolarity values are restored within 1–2 min after instillation [69]. The level of hypotonicity may be crucial in determining therapeutic outcomes. While no significant difference was observed between moderately hypotonic (215 mOsm/L) and isotonic (305 mOsm/L) sodium hyaluronate solutions [70], further reduction in osmolarity to 150 mOsm/L significantly improved therapeutic outcomes [68,71]. A placebo-controlled clinical study performed in 444 subjects using a patented formulation of 0.18% sodium hyaluronate with an osmolarity of 150 mOsm/L has also shown a statistically significant reduction in DED symptoms [72]. Although no adverse effects were observed in this study, recent concerns over the potential of hypotonic solutions to cause microcystic corneal oedema to warrant further long-term safety evaluations [14].

More recently, “osmoprotectants”, which are naturally occurring small molecules that purportedly reduce the concentration of intracellular organic salts without disturbing cellular macromolecular components, have also been investigated for their potential to reduce tear fluid hyperosmolarity [14,73]. Osmoprotectants may be weak polyelectrolytes, such as carbohydrates (e.g., trehalose and hypromellose) and polyols (e.g., glycerol, erythritol, inositol and sorbitol), or zwitterions, such as amino acids (e.g., glycine, betaine, proline, taurine) and methylamines/methylsulfonium solutes (e.g., l-carnitine), that function by a variety of mechanisms to prevent cell death and reduce inflammation [74,75,76]. Erythritol and l-carnitine are currently used as osmoprotectants in Refresh Optive^®^ lipid based eyedrops (Allergan, Irvine, CA, USA), which reportedly improves DED symptoms due to the combined effect of lipid and osmoprotectant [77,78]. A 3% trehalose solution, marketed as Thealoz^®^ (Théa Laboratories, Clermont-Ferrand, France) can also significantly improve DED symptoms [79] and has shown prophylactic benefits in preventing DED secondary to laser eye surgery [80].

To prolong the symptomatic relief provided by eyedrops, artificial tear supplements are often formulated with viscosity-building macromolecules, such as sodium hyaluronate (e.g., Hylo-Vision^®^ HD Eye Drops, OmniVision GmbH, Puchheim, Germany), polyethylene glycol (e.g., Blink^®^ Tears, Abbot Vision, Green Oaks, IL, USA), carboxymethyl cellulose (e.g., Refresh Liquigel^®^ Drops, Allergan, Irvine, CA, USA), polyvinylalcohol (e.g., Blink^®^ Refreshing, Abbot Vision, Green Oaks, IL, USA), carbomers (e.g., Artelac^®^ Nighttime Gel, Bausch & Lomb, Rochester, NY, USA) and natural gums (e.g., Systane^®^, Alcon, Fort Worth, TX, USA) [81]. Hydroxypropylcellulose ophthalmic inserts (Lacrisert^®^; Bausch & Lomb, Rochester, NY, USA) that are intended to be placed in the cul-de-sac and slowly dissolve in the tear fluid may be recommended in moderate-to-severe dry eye to provide sustained lubrication [82,83]. In addition to prolonging the precorneal residence time, viscosity-building polymers also exhibit mucomimetic properties and form a protective layer on the ocular surface reducing surface desiccation, friction and epithelial cell death [84]. However, blurring of vision and difficulty in instillation are major drawbacks of viscous solutions. In situ gelling bioadhesive systems based on hydroxypropyl guar (HP-Guar Systane^®^, Alcon, Fort Worth, TX, USA) which form a soft gel at physiological tear pH (approximately 7.4) have thus been developed to minimise dosing difficulties [85].

#### 3.1.2. Lipoidal Tear Supplementation

Excessive evaporation of tears due to a compromised or absent lipid layer is a hallmark characteristic of EDE [86], often associated with MGD. Patients with a baseline lipid layer deficiency generally observe greater benefit with lipoidal tear supplementation [87] as these eyedrops replenish the tear fluid lipid layer, potentially improving its stability and reducing evaporation of the underlying aqueous phase [65,88]. Consequently, lipid-based eyedrops can also help in relieving DED symptoms in the presence of desiccating stress [89]. Lipid-based (lipomimetic) eyedrops are most frequently available in the form of oil-in-water emulsions (e.g., Cationorm^®^, Santen SAS, Évry Cedex, France), or ointments (e.g., VitA-POS^®^, AFT Pharmaceuticals, Auckland, New Zealand). These eyedrops usually contain amphipathic lipids which reduce the interfacial tension between the aqueous and lipid components of the tear film to improve tear film stability and continuity [90,91].

Polar lipids, such as phospholipids and ω-hydroxy fatty acids, although present in a relatively small concentration in the tear film, play a critical role in determining its surface properties and stability. It has been suggested that polar lipid abnormalities may have aetiological roles in EDE [92]; hence, lipomimetic eyedrops are often formulated with phospholipids. Alcon’s Systane Balance, for instance, is based on the patented LipiTech™ system and is a microemulsion of mineral oils and a polar phospholipid surfactant (phosphatidylcholine) specifically designed to minimise the evaporative loss of tears from the ocular surface [91]. Studies comparing Systane Balance to the non-lipid containing Systane Ultra showed that the lipomimetic eyedrop provides a more consistent barrier to excessive tear evaporation and has superior prophylactic efficacy in an adverse environment [93]. Similar prophylactic benefits in a simulated adverse environment have also been observed with Systane Complete [94], which too is based on the LipiTech system but contains nano-sized lipid droplets that are anticipated to have better patient tolerance due to lower opacity.

Liposomal sprays, such as ActiMist™ (Optrex Ltd., Berkshire, UK) and Tears Again^®^ (Optima Pharmazeutische GmbH, Hallbergmoos, Germany) also contain a relatively large proportion of phospholipids, which can accumulate at the interface of the aqueous and lipoidal phase of the tear fluid and improve its integrity [95,96]. An in situ gelling hybrid artificial tear supplement with liposomes containing phosphatidylcholine, cholesterol, vitamins A and E, suspended in an aqueous phase consisting of gellan gum and the osmoprotectants l-carnitine and trehalose has also been designed to replenish both aqueous and lipid components of the tear film [97].

**Figure 3:**
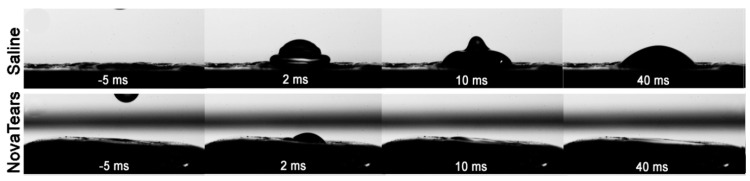
Representative images comparing the spreading dynamics of saline and semifluorinated alkane eyedrops on the corneal surface under typical conditions for dispensing eyedrops. Adapted with permission from [38]; Published by Elsevier, 2019.

SFAs are synthetic pharmacologically inert hydrophobic liquids that reportedly stabilize the tear film lipid layer. Due to their unique surface properties and extremely low surface tension, SFAs spread rapidly on the ocular surface with their contact angle on the cornea being virtually zero (Figure 3), thus they have the potential to improve continuity and integrity of the tear film and fortify the barrier to tear evaporation [38].

The SFA, perfluorohexyloctane, is currently marketed as a lipid layer stabilizing eyedrop (EvoTears™, URSAPHARM in Europe and NovaTears™, AFT Pharmaceuticals in Australia and New Zealand). Based on the patented EyeSol^®^ technology (Novaliq GmbH, Heidelberg, Germany), this eyedrop is preservative-free and is believed to have slower precorneal clearance than aqueous eyedrops. Preclinical and clinical studies have shown that NovaTears can improve lipid layer thickness and integrity and minimise tear fluid evaporation, thus improving clinical signs of mild to moderate EDE associated with MGD [98,99].

As discussed previously, considerable overlap exists between the various subtypes of DED and it is not uncommon to see features of aqueous and lipid deficient DED simultaneously [2]. Scifo et al. [100] compared the efficacy of lipid-based eyedrop Emustil^®^ (SIFI, Aci Sant’Antonio, Italy), which is an oil-in-water emulsion containing soybean oil and egg yolk phospholipids, and aqueous sodium hyaluronate eyedrops in a mouse model of DED. They reported a significant improvement in tear volume after administration of the lipid-based eyedrop but not with the aqueous eyedrops. However, when the two eyedrops were administered concomitantly, improvement in clinical signs of DED was observed more rapidly, thus demonstrating the rationale for concomitant administration of aqueous and lipid-based eyedrops when both evaporative and the aqueous-deficient components of DED are present.

### 3.2. Medicated Topical Ophthalmic Formulations

**Figure 4:**
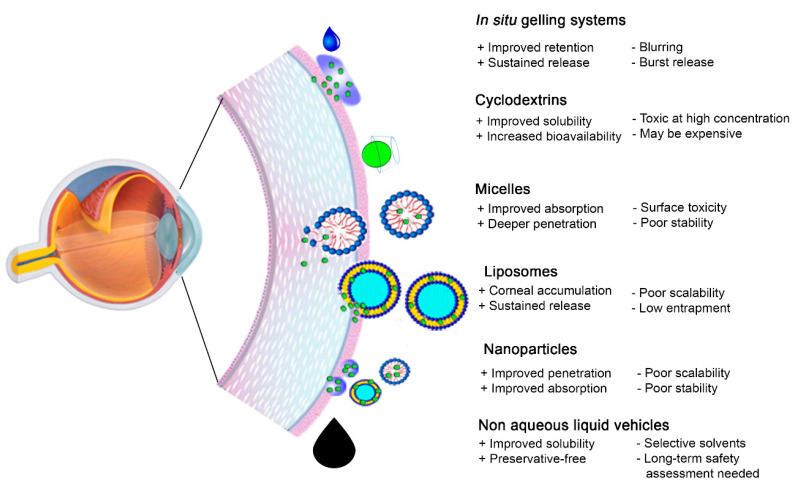
Schematic representation of novel ophthalmic dosage forms used to enhance topical drug delivery for DED therapy. Adapted with permission from [102]; Published by Elsevier, 2016.

Medicated ophthalmic formulations may be considered to manage chronic DED when artificial tear supplements are insufficient (Figure 2 and Table 1). Colloidal drug delivery systems, such as liposomes, nanoparticles and micelles, as well as non-aqueous vehicles have thus been researched to solubilize lipophilic drugs and enhance ocular drug penetration (Figure 4). The poor aqueous solubility of lipophilic drugs has been a persistent challenge in topical formulation development, which may be further exacerbated by precipitation of the dissolved drug due to dilution and washout by the tear fluid [101]. Drug precipitation may also be observed on concomitant administration of more than one eyedrop due to a rapid change in the immediate environment of the drug. The formulation strategies typically used to enhance ocular drug delivery and their application in the management of DED are briefly discussed below.

#### 3.2.1. Cyclodextrins

Cyclodextrins (CDs) are increasingly being utilised in ocular drug delivery due to their potential for drug solubilization and stabilization. CDs are cyclic oligosaccharides of 6 (α-CD), 7 (β-CD), or 8 (γ-CD) glucopyranose units with a hydrophobic cavity surrounded by a hydrophilic outer surface. In aqueous solutions, water from the hydrophobic cavity is displaced by hydrophobic drug molecules to form large water-soluble complexes. CDs have frequently been used in the preparation of aqueous cylosporine A (CsA) eyedrops with significant improvement in transcorneal penetration and retention [102]. Hydroxypropyl-ß-CD has also been used for ocular delivery of tacrolimus [103] and loteprednol etabonate [104] and was found to significantly improve their ocular bioavailability.

The unique solubilizing characteristics of specific CDs have also been harnessed in the preparation of solubilizing nanoparticles. Solubilising nanoparticles contain drug-CD complexes that form nanoparticles in aqueous solution, enhancing drug solubility in the aqueous tear fluid [105]. The drug-CD complexes also penetrate the mucus layer and provide sustained drug release improving drug permeation and bioavailability within the ocular tissues. This technology has been used by Oculis Pharmaceuticals (Lausanne, Switzerland) to formulate eyedrops containing a novel compound named OCS-02, an anti-TNFα antibody specifically designed for topical delivery. Clinical trials evaluating safety and efficacy suggest a promising profile for treating ocular inflammatory conditions, including uveitis and DED [106].

#### 3.2.2. Micelles

Micelles are self-assembling spherical colloidal systems formed by the orientation of surfactant molecules to form a hydrophobic core enclosing the drug within an outer hydrophilic shell. Nano-sized micellar systems have shown increased uptake in all layers of the cornea [107]. Several micellar formulations of CsA have been registered and approved. Papilock mini^®^ (Santen SAS, Evry Cedex, France) is a 0.1% micellar CsA formulation approved for the treatment of vernal keratoconjunctivitis sicca in Japan in 2005 while TJ Cyporin^®^ (Taejoon Pharmaceutical Co., Seoul, Korea) was approved in 2003 for DED treatment in South Korea. Modusik-A Ofteno^®^ (Laboratories Sophia, Guadalajara, Mexico), based on the Sophisen^®^ delivery platform, contains 0.1% CsA and has been approved for DED treatment in Latin America [101]. The Sophisen drug delivery platform has also been used to develop diclofenac sodium eyedrops (3-A Ofteno^®^) for the management of ocular inflammation [108]. Meanwhile, Cequa^®^ (formerly Seciera™, Sun Pharma, Mumbai, India) is the first micellar formulation approved by the FDA for DED. This preservative-free 0.09% formulation of CsA has shown good safety and efficacy in clinical trials and offers the advantages of improved stability, fewer manufacturing deadlocks and thus greater cost-effectiveness compared to most other colloidal CsA formulations [109].

#### 3.2.3. Liposomes

Liposomes are membrane-like spherical vesicles composed of phospholipid bilayers that can solubilize both hydrophilic and hydrophobic drugs. The phospholipid component of liposomes may also have a role in stabilizing the lipid layer of the tear film, as discussed above, rendering them particularly advantageous for DED therapy. Hence, liposomes have been investigated for ocular delivery of several DED drugs, such as CsA [110], medroxyprogesterone acetate [111] and sirolimus [112]. However, in practice, liposomes have poor stability on the corneal surface and provide only moderate improvement in bioavailability over traditional ophthalmic eyedrops [113]. Poor shelf-life, poor entrapment efficacy and difficulty in manufacturing sterile formulations are major limitations in the development of liposomal formulations. CsA proliposomes, which are dry, free-flowing powders that spontaneously assemble in situ to form liposomes, have therefore been developed to counter some of these limitations. Proliposomes are easier to scale-up and have a longer shelf-life; however, their safety and efficacy in vivo are yet to be demonstrated [114].

#### 3.2.4. Nanoformulations

Nanoparticles and nanoemulsions are often used to enhance topical drug delivery as their small size and customizable surface properties can enhance drug uptake into the cornea [115,116]. In 2003, Restasis, a preservative-free anionic oil-in-water nanoemulsion, was the first FDA-approved CsA formulation to be introduced to the US market, while Lacrinmune^®^ (Bausch and Lomb, Rochester, NY, USA) nanoemulsion was licensed in Argentina. The Lacrinmune formulation is almost identical to Restasis, except that it also contains sodium hyaluronate to enhance precorneal residence. A cationic nanoemulsion containing double the CsA concentration launched by Santen in Europe in 2015 under the tradename Ikervis^®^, has shown better ocular tolerability as well as higher CsA bioavailability than Restasis [117,118].

To prolong ocular residence, cationic nanoparticles are often utilised to improve mucoadhesion by ionic interaction of the positively charged particles with negatively charged sialic acid residues in the mucus. CsA nanoparticles surface-modified with phenylboronic acid have shown increased ocular retention within in vivo studies [119]. This study suggested that therapeutically relevant drug concentrations in the eye may be maintained even with once-weekly administration. Another approach involves mucus penetrating nanoparticles that have been developed to reduce particle entrapment and increase ocular bioavailability [120]. This technology has been used to formulate loteprednol etabonate nanoparticles coated with Poloxamer 407 (EYSUVIS^TM^, Kala Pharmaceuticals, Waltham, MA, USA) [121], which received FDA approval for short-term (up to two weeks) treatment of DED signs and symptoms on 27 October 2020.

As with most colloidal systems, poor stability and scalability are major limitations of nanoparticles since excipients, solvents, as well as manufacturing and sterilization methods, often need to be adapted for large-scale production affecting the cost and acceptability of the finished product [116]. Phase-sensitive in situ precipitating nanoparticles have thus been developed to improve shelf-life and manufacturability [122]. Ocular bioavailability of these in situ precipitating nanoparticles was found to be several times greater than that of Restasis. However, due to low moisture levels and tear fluid imbalance in DED, in situ precipitation may be unreliable and their irritation potential may be high. Solid lipid nanoparticles, in which the drug is solubilised in lipids that are solid at room temperature, have also been developed to improve formulation shelf-life and overcome the technical difficulties typically encountered in commercialising nanoformulations. Solid lipid nanoparticles prepared using glyceryl behenate as the lipid matrix have shown improved CsA bioavailability in vivo [123], although no solid lipid nanoparticle formulation has made it to the market yet.

A major concern regarding the application of nanoformulations in ocular drug delivery is the use of high surfactant and co-surfactant concentrations which increase the potential for ocular irritation and, paradoxically, may result in iatrogenic DED [51]. It cannot be overlooked that while having the benefit of improved penetration and efficacy, the small size of nanomaterials also risks an increased toxicity potential due to increased cellular interactions [116]. In vivo studies performed using a modified Draize test have shown that for several excipients, ocular toxicity may be evident when used in nanomaterials but not when formulated as conventional eyedrops [124].

#### 3.2.5. Non-Aqueous Vehicles

Application of non-aqueous vehicles in ocular drug delivery is relatively limited, primarily because their long-term safety and biocompatibility are poorly understood. Vegetable oils were historically used to prepare oily eyedrops; however, the high viscosity and refractive index of oily liquids tend to increase patient discomfort due to vision blurring and foreign body sensation. Moreover, vegetable oils can cause corneal toxicity and related adverse effects [125,126]; hence, their use as eyedrop vehicles is now limited.

Of late, some non-aqueous vehicles, such as SFAs, have found application in DED management since they can be used to prepare preservative-free eyedrops (NovaTears and EvoTears) that reportedly stabilise the tear film lipid layer. Due to their low refractive index, SFAs have previously been used as vitreous substitutes [127] and as intra-operative tools in retinal translocation procedures [128,129]. As such, their biocompatibility in the ocular environment is well established. The low refractive index of SFAs (1.32–1.34) is also beneficial for topical application since the blurring of vision is minimised [42]. SFAs have also been used as vehicles for ocular drug delivery and a CE Mark has now been assigned to SFA based Omega-3 eyedrops (EvoTaers/NovaTears Omega). SFAs are also being tested for ocular delivery of CsA (CyclASol^®^, Novaliq GmbH, Heidelberg, Germany) with ex vivo studies showing improved corneal bioavailability with SFA based formulations in comparison to Restasis and Ikervis [130]. Meanwhile, phase II clinical trials of CyclASol have proven good efficacy, safety, and tolerability with a positive benefit-to-risk ratio [131]. Moreover, azithromycin suspensions prepared in SFAs showed good conjunctival absorption and antibacterial activity in lid tissues colonised with bioluminescent *Staphylococcus aureus* bacteria ex vivo [132].

Medium-chain triglycerides are frequently used as the oil phase in emulsions intended for ocular administration. In 2012, Azyter^®^ (Laboratories Thea, Clermont-Ferrand, France), a 1.5% *w*/*w* azithromycin eyedrop prepared using medium-chain triglycerides as the vehicle was approved in Europe for the treatment of bacterial conjunctivitis [133]. Azyter is relatively well-tolerated with minimal irritation and was found to be an effective treatment option for chronic blepharitis [134].

A major advantage of non-aqueous vehicles in ocular drug delivery is their potential to solubilize hydrophobic drugs without the addition of surfactants. Furthermore, due to the inability of bacteria to proliferate in the absence of water, no preservatives are needed [132], reducing the risk of iatrogenic DED.

## 4. Concluding Remarks

Although significant research has been performed in the field of ocular therapies over the past decades, advancement in topical ocular formulations has been rather restrained as formulation scientists still face the dual challenge of circumventing the formidable anatomical and physiological barriers of the eye while also ensuring that there is no permanent damage to the ocular tissues. Ironically, formulation strategies that significantly improve bioavailability usually also worsen the toxicity potential by increasing exposure, while drug penetration also increases with higher ocular toxicity due to the compromised ocular barrier function. Consequently, the ideal topical drug delivery system seeks to achieve the best compromise between safety and efficacy. This is especially the case in the management of ocular surface disorders, such as DED, since the integrity of the ocular surface and tear film are already compromised, increasing the risk of toxic reactions and adverse events. Moreover, with the challenges of treating DED, frequent eyedrop administration over prolonged periods is often required, thus increasing the toxicity potential.

Due to the chronic multifactorial nature of DED, formulations that can simultaneously target multiple underlying pathologies, as appropriate, are desirable. Combination formulations, such as those that lubricate the ocular surface to provide symptomatic relief while also delivering therapeutic agents to the eye, ideally without the need for preservatives, have the potential to improve the treatment safety and efficacy by reducing the washout effect as well as the overall excipient exposure. Such combination eyedrops are likely to show a faster onset of action and may potentially reduce the cost and inconvenience of treatment, thus facilitating better patient compliance and treatment outcomes.

## Figures and Tables

**Table 1:** Staged management and treatment recommendations of DED.

**Stage 1**	
Education	Provide information on the condition, its management, treatment and prognosis
Environmental modification	Minimize exposure to high temperature/low-humidity environments and air conditioning/forced hot-air systems; use humidifiers and air filters indoors; avoid exposure to pollutants, volatile organic compounds and wind drafts
Lifestyle modification	Lower video display terminals to below the eye level; take periodic breaks; increase blink frequency (blinking exercises); avoid smoking and alcohol consumption
Dietary changes	Increase dietary intake of omega-3 essential fatty acids
Medication review	Identify and potentially modify/eliminate any offending systemic and topical medications (e.g., antihistamines, antidepressants, anxiolytics, oestrogen-containing hormone replacement therapy)
Lid hygiene	Apply warm compresses and lid massage
Tear supplementation	Apply low viscosity eyedrops; consider lipid-containing eyedrops for patience with evidence of MGD
**Stage 2 (If Above Options Are Inadequate or Insufficient):**	
Tear supplementation	Apply low to moderate viscosity preservative-free eyedrops to minimise preservative-induced toxicity
Tear conservation	Use moisture chamber spectacles/goggles and/or punctal occlusion (collagen plugs-short term/silicone plugs-long term)
In-office treatments (for MGD)	Apply heat and express meibomian glands (including device-assisted therapies, such as LipiFlow); use intense pulsed light
Prescription medicine	Consider one or more of the following: Topical antibiotic or antibiotic/steroid combination applied to the lid margins for anterior blepharitis (if present) Topical secretagogues (such as diquafosol) Topical corticosteroid (short-term) and non-glucocorticoid immunomodulatory drugs (such as cyclosporine A) Topical LFA-1^#^ antagonist drugs (such as lifitegrast) Oral macrolide or tetracycline antibiotics Oral omega-3 fatty acid supplements
**Stage 3 (If Above Options Are Insufficient or Inadequate):**	
Tear stimulation	Take oral secretagogues
Biological tear substitutes	Apply autologous/allogeneic serum eyedrops
Therapeutic contact lenses	Use soft bandage lenses; rigid mini-scleral and scleral contact lenses
**Stage 4 (If Above Options Are Insufficient or Inadequate):**	
Prescription medicine	Use topical corticosteroid (for longer duration) and systemic anti-inflammatory agents
Surgical intervention	Consider punctal cautery; amniotic membrane grafts; tarsorrhaphy; salivary gland transplantation

Table adapted from the report of the TFOS DEWS II Management and Therapy Subcommittee [62] and the report of the Management and Treatment Subcommittee, International Workshop on Meibomian Gland Dysfunction [63]. LFA-1^#^: Lymphocyte function-associated antigen 1.

## Data Availability

Not applicable.
